# Recent Progress of Novel Nanotechnology Challenging the Multidrug Resistance of Cancer

**DOI:** 10.3389/fphar.2022.776895

**Published:** 2022-02-14

**Authors:** Chengyuan Zhang, Xuemei Zhou, Hanyi Zhang, Xuanliang Han, Baijun Li, Ran Yang, Xing Zhou

**Affiliations:** ^1^ School of Pharmacy and Bioengineering, Chongqing University of Technology, Chongqing, China; ^2^ Chongqing Key Laboratory of Medicinal Chemistry and Molecular Pharmacology, Chongqing University of Technology, Chongqing, China; ^3^ Department of Pharmacy, Chongqing Hospital of Traditional Chinese Medicine, Chongqing, China

**Keywords:** nanotechnology, multidrug resistance, cancer, mechanism, review

## Abstract

Multidrug resistance (MDR) of tumors is one of the clinical direct reasons for chemotherapy failure. MDR directly leads to tumor recurrence and metastasis, with extremely grievous mortality. Engineering a novel nano-delivery system for the treatment of MDR tumors has become an important part of nanotechnology. Herein, this review will take those different mechanisms of MDR as the classification standards and systematically summarize the advances in nanotechnology targeting different mechanisms of MDR in recent years. However, it still needs to be seriously considered that there are still some thorny problems in the application of the nano-delivery system against MDR tumors, including the excessive utilization of carrier materials, low drug-loading capacity, relatively narrow targeting mechanism, and so on. It is hoped that through the continuous development of nanotechnology, nano-delivery systems with more universal uses and a simpler preparation process can be obtained, for achieving the goal of defeating cancer MDR and accelerating clinical transformation.

## Introduction

Cancer is still a worldwide malignant disease. According to the latest statistics of CA 2021 (CA: A Cancer Journal for Clinicians), by 2020, the number of cancer deaths in the world had reached 9.96 million (Sung et al., 2021). With the developments in cancer pathology, the research on chemotherapeutic drugs developed for cancer has obtained tremendous advances. However, due to its inherent characteristics, chemotherapy still faces some challenges, such as lack of sufficient targeting ability and limited bioavailability (Subramanian et al., 2016; Rodriguez-Nogales et al., 2018; Mu et al., 2020). More importantly, repeated treatments of chemotherapeutics lead to multiple drug resistance of tumor cells (Kibria et al., 2014) so that patients with malignant tumors (such as non–small cell lung cancer and triple-negative breast cancer) may initially respond to the first-line chemotherapy strategy; their tumor growth is obviously inhibited, but they often have cancer metastasis or recurrence; then, second-and third-line chemotherapy or other treatments are needed (Wu et al., 2010; Shapira et al., 2011; Brufsky et al., 2012). Consequently, the response of tumor cells to the subsequent chemotherapy using various cytotoxic drugs becomes ineffective. Therefore, finding a broad and effective treatment strategy to reverse multidrug resistance is a very important topic in tumor treatment research.

**FIGURE 1:**
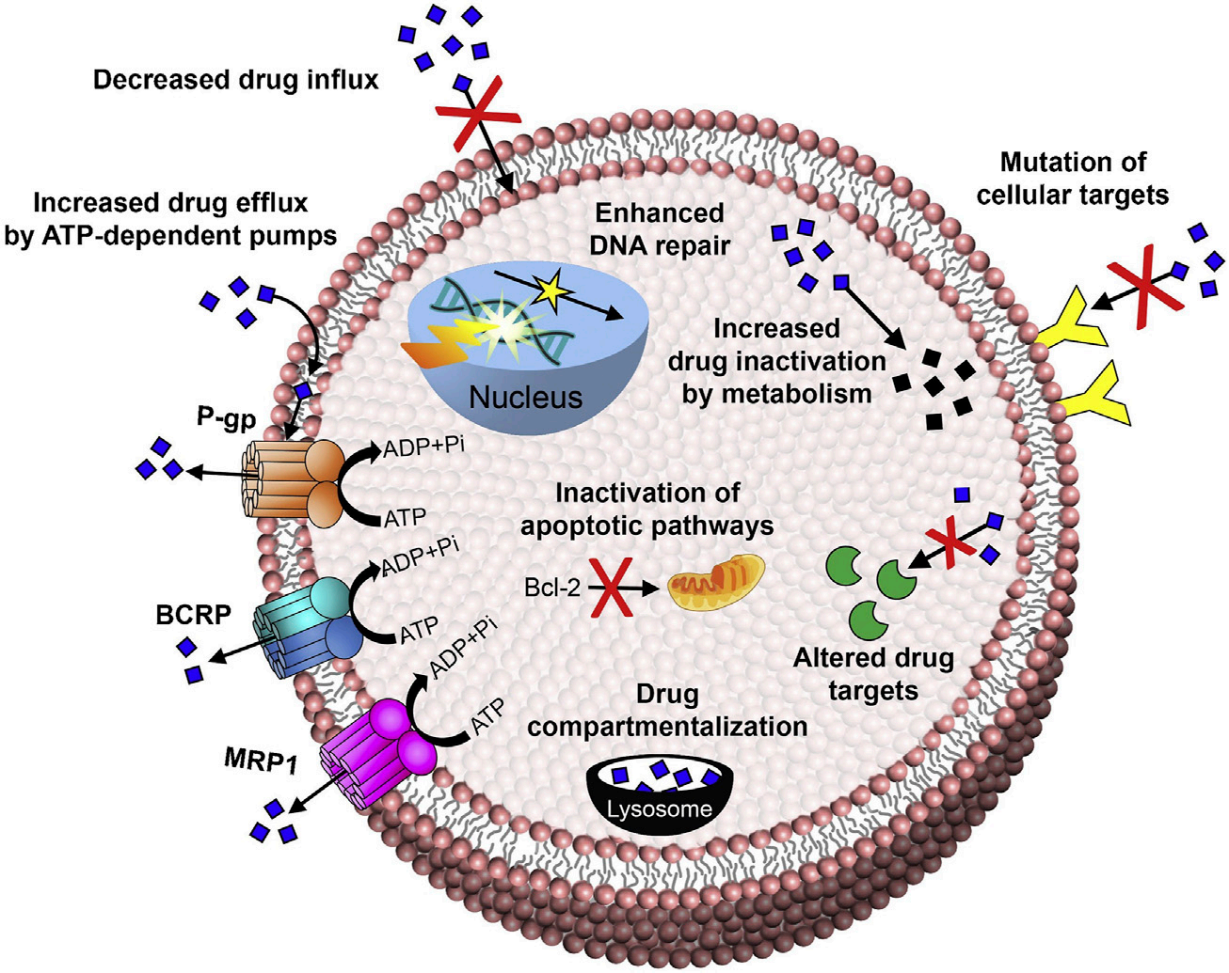
Mechanisms of anticancer drug resistance: efflux pump-mediated mechanisms of MDR and efflux pump-independent drug resistance mechanisms (Bar-Zeev et al., 2017). Copyright 2017 Elsevier Ltd. This figure was generated by Microsoft PowerPoint and OneKeyTools Lite.

It has been proven that the mechanisms of tumor multidrug resistance mainly include ATP-dependent drug efflux, DNA repair, inhibition of the apoptosis pathway, and tumor tissue heterogeneity in recent years (Figure 1) (Shapira et al., 2011; Housman et al., 2014; Bar-Zeev et al., 2017; Wei et al., 2021). In addition to that, the hypoxic tumor microenvironment (Zeng et al., 2015; Jing et al., 2019), cancer stem cell regulation (Donnenberg and Donnenberg, 2005; Mihanfar et al., 2019), endoplasmic reticulum stress (Bahar et al., 2019), and the immune suppression microenvironment of tumors (Vasan et al., 2019) were also proven to be closely related to the progress of MDR. At present, although the severity of multidrug resistance can be alleviated by various therapeutics, which can mainly increase the sensitivity of tumor cells to chemotherapeutic drugs by reducing the drug efflux of tumor cells, these small molecular drugs still have the same disadvantages as mentioned above, such as lack of targeting and bioavailability (Milane et al., 2011; Gao et al., 2012). More importantly, it is difficult for these drugs to approach the tumor site along with chemotherapy drugs simultaneously, for achieving an ideal synergistic effect and eventually a better clinical treatment effect (Duan et al., 2013; Meng et al., 2013; Zhang M. et al., 2017; Wang and Huang, 2020).

With the continuous development of nanotechnology, rationally designed nanoscale drug delivery systems (NDDSs) with their distinct advantages can be employed to deliver various bioactive payloads to tumor sites for resolving the aforementioned problems (Acharya and Sahoo, 2011; Bertrand et al., 2014; Guo and Huang, 2020; Xu et al., 2021). Through evading or inhibiting tumor MDR mechanisms, NDDSs can effectively improve the enrichment process of chemotherapeutics, or sensitivity of tumor cells, to obtain obvious therapeutic effects (Lim et al., 2019; Wang L. et al., 2019; Ji C. et al., 2021). Therefore, the development of more ideal NDDSs, which can synergistically reverse multidrug resistance, has become one of the important topics in the field of tumor therapy. This review will focus on the different mechanisms of multidrug resistance and systematically summarize the relevant progress of NDDSs which are utilized for synergistically treating tumors effectively and reversing multidrug resistance in recent 3 years, to provide references for exploring the next generation NDDSs targeting MDR.

## NDDSs Targeting Drug Efflux Pumps for Inhibiting MDR

As illustrated by Figure 1, among these mechanisms involved in MDR, the drug efflux mechanism is the most important one which obtains the most common and intensive research. After continuous stimulation by chemotherapeutic drugs, tumor cells will upregulate drug efflux–related transporters, especially including multidrug resistance protein 1 (MDR1), multidrug resistance–associated protein 1 (MRP1), and breast cancer resistance protein (BCRP) (Shapira et al., 2011; Housman et al., 2014). The physiological processes of these transporters are ATP energy-dependent, so they belonged to the ATP-binding cassette (ABC) transporter family. After recognizing chemotherapeutic drugs with a molecular weight less than 2,000 Da, ABC proteins will pump these drugs out immediately (Ambudkar et al., 2003). As a result, the intracellular drug concentration will be reduced significantly, with decreased or even completely lost chemotherapeutic efficiency. Therefore, targeting the ABC proteins and engineering a novel kind of NDDS for regulating or evading the MDR pathway has become an important research topic for reversing the MDR of tumors.

### NDDSs Reducing the Content of MDR-Related Proteins

**FIGURE 2:**
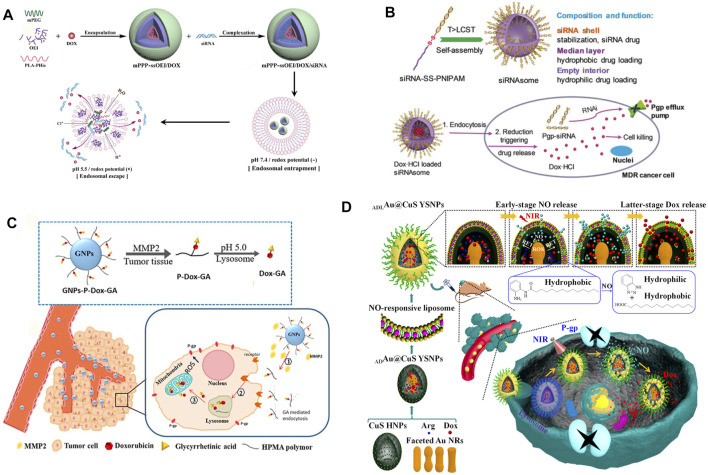
**(A)** Schematic illustration of construction and effective endo-lysosomal escape of the pH/redox dual-responsive mPPP-ssOEI/DOX/siRNA co-delivery polyplex. After being internalized, the acidic and reduction potential environment of the endo-lysosome triggers the payload release and the subsequent endo-lysosomal escape (Gao et al., 2019). Copyright 2019 American Chemical Society. **(B)** Illustration of the formation, composition, and reservation progress against MDR tumor cells of siRNAsome (Zheng et al., 2019). Copyright 2019 John Wiley & Son, Inc. **(C)** Schematic illustration of entry into tumor cells of GNPs-P-DOX-GA and its efficient targeting regulation to mitochondria under multi-stimuli (Liu Z. et al., 2019). Copyright 2019 American Chemical Society. **(D)** Illustrating the NO and DOX programmable release and MDR cancer therapy of ADLAu2@CuS YSNPs (Wang L. et al., 2019). Copyright^©^ 2019 American Chemical Society. This figure was generated by Microsoft PowerPoint and OneKeyTools Lite.

MDR1 (also known as P-glycoprotein, P-gp) is highly expressed in a variety of drug-resistant tumor cells, which are mainly distributed on the surface of the cell membrane. It is a typical ATP-dependent protein and plays a direct important role in the efflux of small molecule drugs (Ghetie et al., 2004; Housman et al., 2014; De Vera et al., 2019). In recent years, RNA interference (RNAi) technology has been employed to inhibit the expression of related proteins. It has achieved amounts of promising results in tumor therapy, especially in the process of reversing multidrug resistance (He et al., 2019; Chen Q. et al., 2020; Ge et al., 2020). NDDSs have proven the ability to protect small interfering RNA (siRNA) from degradation in the process of circulation (Dong et al., 2019b). In addition, NDDSs can load chemotherapy drugs and siRNA together to simultaneously transport them to the tumor site for obtaining enhanced combination therapeutic effects for reversing multidrug resistance and inhibiting tumor growth (Gao et al., 2019; Mendes et al., 2019; Wang C. et al., 2020; Zhu et al., 2020). For example, a novel kind of dual-responsive polyplex, a complex consisting of a variety of polymers, with effective endo-lysosomal escape composed of methoxy poly (ethylene glycol)-polylactide-polyhistidine-ss-oligoethylenimine (mPEG-b-PLA-PHis-ssOEI) was developed by Gao, Y. et al., which was employed for co-delivering MDR1 siRNA and doxorubicin (DOX) (Figure 2A). Meanwhile, the payloads were triggered a release in response to pH/redox stimuli, due to the pH-sensitive poly (l-histidine) (PHis) protonation and the disulfide bond cleavage. The polyplex provided a much higher payload delivery efficiency, MDR1 gene silence efficiency, cytotoxicity against MCF-7/ADR cells (adriamycin-resistant human mammary adenocarcinoma), and stronger MCF-7/ADR tumor growth inhibition (Gao et al., 2019). Another example is a particular kind of all-in-one fluorescent silicon nanoparticle (SiNP)-based NDDS described by Guo, D. et al. This novel NDDS was engineered for visible co-delivering P-gp siRNA and DOX. This approach enhanced therapeutic efficacy in multidrug-resistant breast cancer cells. Notably, this NDDS enhanced the stability of siRNA in the biological environment and entitled the loaded siRNA with a responsive release behavior. As a result, the expression of P-gp was downregulated by approximately 80%. The results of the cell survival experiment indicated that this co-delivery nanoscale delivery system obtained the pronounced therapeutic efficiency to MDR cancer cells (Guo et al., 2019).

The traditional gene delivery system needs to use several positive charge carriers, which bring a huge risk of toxicity *in vivo* (Lv et al., 2006; Wang et al., 2014). Therefore, researchers recently began to employ the regulatory gene itself as a part of the carriers to construct NDDSs, for delivering synergistic chemotherapeutics and achieving ideal safety and therapeutic effect simultaneously (Zheng et al., 2019; Ji W. et al., 2021). Zheng, M. et al. utilized a hydrophilic siRNA shell, a thermal- and intracellular-reduction-sensitive hydrophobic median layer, and an empty aqueous interior to construct a siRNA-based vesicle (siRNAsome). When siRNAsomes were loaded with the hydrophilic drug doxorubicin hydrochloride and anti-P-gp siRNA, synergistic therapeutic activity was achieved in MCF-7/ADR tumor cells and a tumor model (Zheng et al., 2019) (Figure 2B). In addition, due to the high biosafety and targeting ability of exosome and biomimetic vesicles, these studies on constructing new NDDSs have developed vigorously in recent years (Wang T. et al., 2019; Liang et al., 2020; Zhao et al., 2020). Wang, T. et al. utilized versatile mimic vesicles derived from aptamer erythrocytes to investigate a novel kind of delivery system for siRNA and DOX to treat MDR tumors. This system could be readily obtained through extruding erythrocyte membranes and had the advantages of biological homogeneity, high output, controllable size, low cost, and excellent biocompatibility. The drug-loaded vesicles could successfully conquer drug resistance through P-gp silencing and synergistically eliminating MDR tumor cells *via* DOX-induced growth inhibition (Wang T. et al., 2019).

At the same time, the influence of physiological properties of the tumor cell internal and external environment on MDR cannot be ignored. By enhancing the level of reactive oxygen species (ROS) or reducing the level of ATP by targeting mitochondria, NDDSs can effectively inhibit the expression of P-gp, reverse multidrug resistance, and significantly increase the concentration of intracellular chemotherapeutic drugs (Liu Y. et al., 2019; Chang et al., 2020; Del Valle et al., 2020; Sui et al., 2020). For instance, Chang, N. et al. engineered a pH/ROS programmed-responsive and self-accelerating drug release NDDS and utilized it for the treatment of multidrug-resistant human colon adenocarcinoma. This NDDS, named as PLP-NPs, was composed of an ROS-sensitive polymeric paclitaxel (PTX) prodrug (DEX-TK-PTX), a PHis, and an ROS-generating agent, β-lapachone (Lapa). PHis protonation facilitated the escape of the PLP-NPs from the lysosome and the release of Lapa under acidic conditions in lysosomes. Attributed to a large amount of ROS generated by Lapa, ATP within tumor cells was consumed, and P-gp production was downregulated consequently. *In vitro* and *in vivo* experiments subsequently confirmed that this novel kind of NDDS successfully obtained tumor-specific cytotoxicity and reversed MDR (Chang et al., 2020). On the other hand, targeting the mitochondria is also an important strategy to reverse multidrug resistance by reducing the expression level of P-gp. Liu, Y. et al. designed a mitochondria-targeting multi responsible NDDS (GNPs-P-DOX-GA) for overcoming MDR through enhanced ROS generation, where increased cellular uptake of drugs and accumulation of it towards the mitochondria were both achieved by glycyrrhetinic acid (GA) (Figure 2C). GNPs-P-DOX-GA nanoparticles could be degraded by tumor extracellular metal matrix protease-2 (MMP2) and release small size P-DOX-GA to facilitate tumor tissue penetration. After internalization by tumor cells, DOX-GA was released through hydrolysis of the hydrazone bond and then efficiently delivered to the mitochondria (Liu Y. et al., 2019).

As an important substance for constructing NDDSs, nitric oxide (NO) is capable of not only directly eliminating tumor cells but also effectively inhibiting the expression of P-gp, to reduce the drug efflux phenomena of MDR tumor cells. It has been widely used in the synergistic treatment of MDR tumors along with chemotherapeutic drugs (Ding et al., 2019; Wang L. et al., 2019; Wei et al., 2019; Wu W. et al., 2020; Wang J. et al., 2021). Considering an NO-stimulated NDDS as an example, it was rationally designed to release NO and DOX with a significant time gap for promoting MDR cancer therapy. Under 808 nm laser irradiation, ROS was generated in the confined space of Au-ADL@CuS YSNPs, and it effectively converted L-Arg into NO consequently (Figure 2D). As the NO release progressed, the NO-responsive liposome layer was deteriorated more severely, allowing DOX to escape. This NDDS could significantly inhibit the P-gp expression and lead to promising therapeutic effects on MCF-7/ADR cancer cells (Wang L. et al., 2019).

### NDDSs Reducing the Drug Efflux Ability of MDR-Related Proteins

Due to the inherent advantages of NDDSs, it can transport a variety of active substances to the tumor site simultaneously, to induce a synergistic therapeutic effect, such as the nano co-delivery of oxaliplatin and folinic acid, achieving synergistic chemo-immunotherapy with 5-fluorouracil, and the co-delivery of siRNAs of two key inflammation-related proteins (p38α MAPK and p65) by novel liposomal delivery (Wang Y. et al., 2020; Guo et al., 2020). Therefore, through rational design, the inhibitors of MDR-related proteins and chemotherapeutic drugs can reach the tumor lesion at the same time *via* NDDSs (Dong et al., 2019a; Huang et al., 2019; Ren et al., 2019; Zhen et al., 2019; Fathy Abd-Ellatef et al., 2020; Shair Mohammad et al., 2020; Wang S. et al., 2021; Pan et al., 2021). Pan, Y. et al. developed cancer stem cell–specific targeted mSiO_2_-dendritic polyglycerol (mSiO_2_-dPG) nanocarriers for simultaneously delivering the chemotherapy drug DOX along with the P-gp inhibitor tariquidar (Tar) for enhanced chemotherapy to overcome MDR in breast cancer stem cells. The mSiO_2_-dPG nanocarriers possessed a high loading capability, excellent pH stimuli-responsive performance, and good biocompatibility. With the help of cancer stem cell–specific targeting and P-gp inhibitor Tar, the accumulation of DOX delivered by the mSiO_2_-dPG nanocarriers could be greatly increased in the drug-resistant three-dimensional mammosphere of breast cancer stem cells, and the chemotherapeutic efficacy against breast cancer stem cells was enhanced (Pan et al., 2021). On the other hand, as mentioned above, targeting the tumor cell mitochondria can reduce the production of ATP. Because the drug efflux function of P-gp is ATP-dependent, the damage to mitochondrial function will also significantly inhibit the function of P-gp (Gu et al., 2019; Wei et al., 2019; Dong et al., 2020; Cheng et al., 2021; Guo et al., 2021). Guo, W. et al. developed a drug delivery system incorporating PTX onto polyethylene glycol–modified and oxidized sodium alginate–functionalized graphene oxide nanosheets. Owing to pH/thermal-sensitive drug release properties, these nanosheets could induce more obvious antitumor effects on gastric cancer, compared to free PTX. With near-infrared-irradiation, these nanosheets could generate excessive ROS, attack the mitochondrial respiratory chain complex enzyme, reduce the ATP supplement for P-gp, and effectively inhibit its efflux pump function (Guo et al., 2021).

**FIGURE 3:**
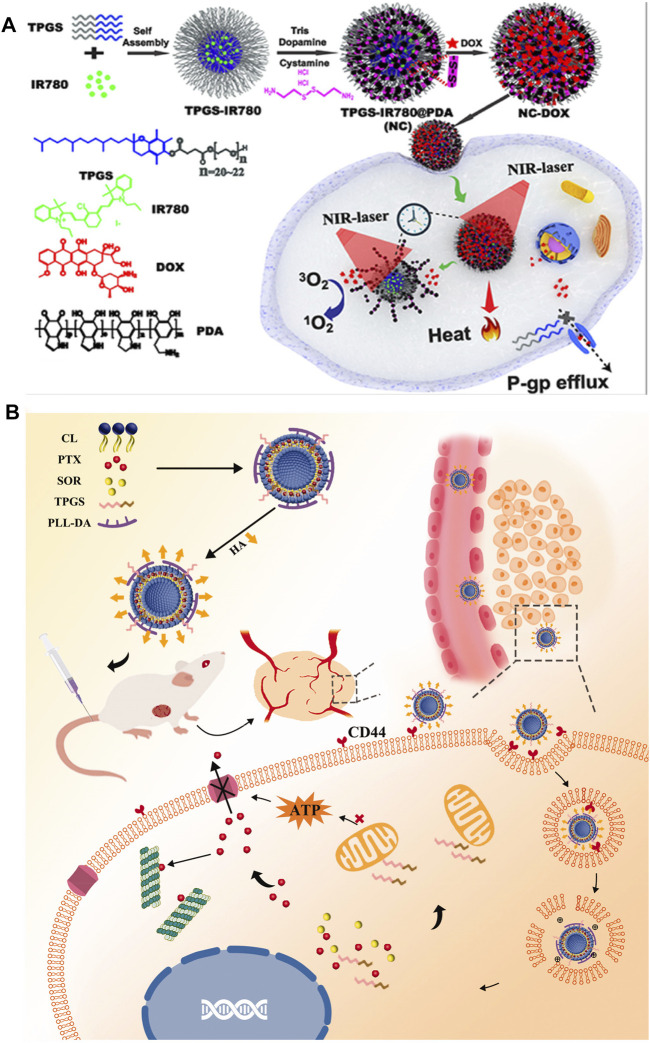
**(A)** Schematic representation of the construction and application of photoresponsive NC with trimodal synergistic therapy properties (Xing et al., 2019). Copyright 2019 American Chemical Society **(B)** Schematic illustration of the HA-TPD-CL-PTX/SOR liposome for co-delivery of PTX and SOR to overcome MDR in cancer cells. Drug delivery includes steps of the intravenous injection, active targeting of liposome, and degradation of HA by HAase together with the exposure of PLL-DA at HAase-rich lysosome, the release of drugs, action on mitochondria function, and inhibition of the P-gp efflux by TPGS to further enhance drug accumulation in cancer cells (Lei et al., 2019). Copyright 2019 Taylor & Francis Group. This figure was generated by Microsoft PowerPoint and OneKeyTools Lite.

In addition, with the deepening and expansion of NDDS engineering research, some carrier molecules with biological regulatory functions have been found, such as D-alpha-tocopheryl poly (ethylene glycol)-1000 succinate (TPGS), which can reduce the ATP content in tumor cells (Lei et al., 2019; Xing et al., 2019; Jiang et al., 2020). Xing, Y. et al. reported a photo-responsive nanocluster system prepared by installing polydopamine nanoparticle clusters on the surface of TPGS micelles solubilized with IR780 (a photosensitizer) to achieve combined chemotherapy/photothermal therapy/photodynamic therapy for drug-resistant breast cancer (Figure 3A). The nanocluster shows prominently quenched fluorescence emission and inhibited singlet oxygen generation upon exposure to near-infrared light, favoring a highly efficient photothermal therapy module. More importantly, TPGS could enhance the intracellular accumulation of doxorubicin hydrochloride whose release is boosted by the photothermal heat. Demonstrated by the results of *in vitro* and *in vivo* experiments, the developed nanocluster exhibited a great potential to treat MDR cancer (Xing et al., 2019). Another example of this special carrier molecule, TPGS, is a multifunctional liposome consisting of TPGS and a polylysine-deoxycholic acid copolymer, paclitaxel, and a chemosensitizing agent, sorafenib. Contributed to the MDR reversal effect of TPGS, this particular kind of liposome significantly increased the cellular concentration of paclitaxel and induced antitumor therapeutic effects (Figure 3B) (Lei et al., 2019).

### NDDSs Designed for Evading Drug Efflux Pumps

MDR relying on the drug efflux mainly realizes the protection of tumor cells by directly reducing the intracellular drug concentration (Housman et al., 2014). Therefore, in addition to directly inhibiting the function and expression of MDR-related proteins, we can also take advantage of the characteristics of the drug efflux process to evade the exclusion process and improve the intracellular concentration of drugs. As mentioned above, P-gp can recognize drugs with a molecular weight below 2,000 Da and pump them out of cells (Ambudkar et al., 2003). Therefore, it can avoid the drug pumping process of tumor cells to a certain extent by simply using the size property of NDDSs (Chen et al., 2019; Wan et al., 2019; Zhao et al., 2019; Song et al., 2020; Cao et al., 2021; Hu et al., 2021). For example, the amphiphilic block copolymer poly (2-methyl-2-oxazoline-block-2-butyl-2-oxazoline-block-2-methyl-2-oxazoline) (P(MeOx-b-BuOx-b-MeOx) was utilized to form a novel nano-micelle for co-delivering paclitaxel (PTX) and alkylated cisplatin. Superior antitumor activity of co-loaded PTX/CP drug micelles compared to single drug micelles, or their mixture, was demonstrated in cisplatin-resistant human ovarian carcinoma A2780/CisR xenograft tumor and multidrug-resistant breast cancer LCC-6-MDR orthotopic tumor models (Wan et al., 2019).

However, since the release process of drugs from ordinary NDDSs is often free diffusion, the intracellular drug consent is difficult to rapidly accumulate to the ideal level for efficient treatment. To solve this problem, great efforts have been continuously put into developing novel kinds of NDDSs from the following three aspects: 1) building a stimuli-responsible NDDS based on the physiological parameters of the tumor microenvironment; 2) constructing an active targeting NDDS to improve the overall drug concentration into tumor cells; 3) according to the specific action mechanism of the drug, targeting its action position and improving the regional drug concentration for achieving the maximum effect.

#### Construction of Stimuli-Responsive NDDSs Based on the Physiological Parameters of Tumor Cells

For approaching the accurate and rapid drug release, reduced toxic side effects, and increased intercellular drug concentration, some tumor microenvironment physiological properties which are different from those of normal tissues, such as pH and enzyme concentration (Llopis-Lorente et al., 2019; Gannimani et al., 2020), have been widely utilized as environmental stimuli for triggering the drug release of NDDSs, and tremendous studies have emerged continuously in recent years (Zhang C. et al., 2017; Che et al., 2019; Liu Z. et al., 2019; Zhi et al., 2019; Wang J. et al., 2020; Kumar et al., 2020; Luo et al., 2020; Mei et al., 2020).

**FIGURE 4:**
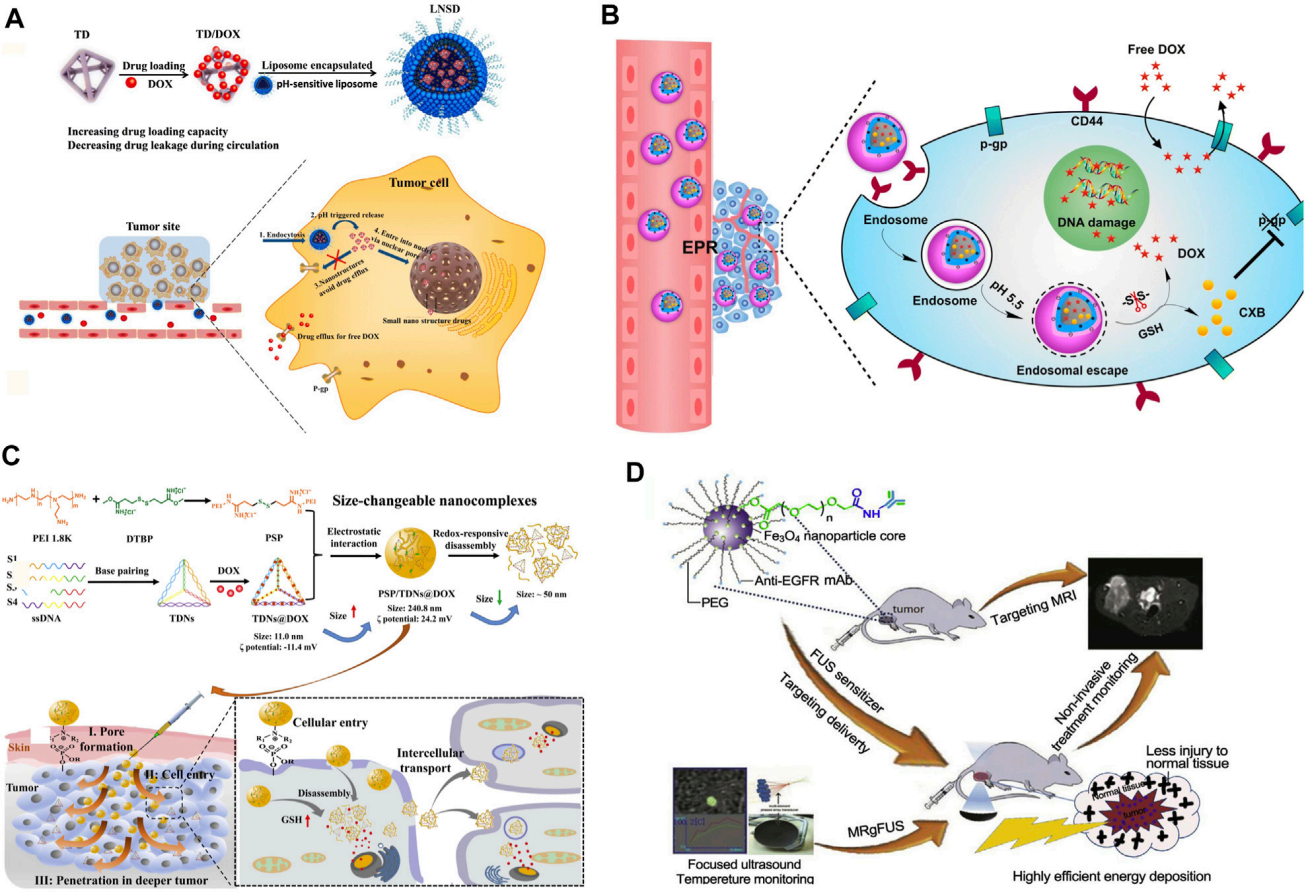
**(A)** Preparation, anti-efflux, and cell nuclear-transport effects of the LNSD (Li et al., 2019c). Copyright 2021 Springer Nature Switzerland AG. **(B)** Schematic illustrations for preparation of HPPDC nanoparticles and their functional mechanisms for overcoming drug resistance in breast cancer treatment (Zhang S. et al., 2019). Copyright 2021 Springer Nature Switzerland AG. **(C)** Schematic representation of the redox-responsive PEI/TDNs/DOX nanocomplexes with membrane-breaking and size-changeable properties for combating MDR tumor (Yan et al., 2021). Copyright 2020 Elsevier B.V. **(D)** Scheme of the formulation process for anti-EGFR-PEG-SPIO NPs and their applications in MRgFUS (Wang et al., 2017). Copyright 2017 Elsevier Ltd. This figure was generated by Microsoft PowerPoint and OneKeyTools Lite.

Among those tumor environmental stimuli, it is the most common strategy to construct responsive nanosystems by using the difference of pH value between the tumor microenvironment and normal tissue. Due to the abnormal metabolism of tumor cells, the pH value of the tumor microenvironment is 6.5–7.0, while the pH values of endosomes and lysosomes are 5–6 and 4.5–5.5, respectively, (Yin et al., 2013; Ghadiri et al., 2017). This special pH gradient can be applied to trigger drug release or dissociation of NDDSs (Wu et al., 2018; Yang et al., 2018; Li et al., 2019c; Wang Q. et al., 2019; Zhang et al., 2020). A pH-sensitive graft copolymer, poly[(3-amino ester)-g-B-cyclodextrin (PBAE-g-(3-CD)], was synthesized by Wang, Q. et al. It was employed to co-deliver DOX and adjudin (ADD, a mitochondrial inhibitor). Triggered by low pH in endo/lysosome after endocytosis, both ADD and hydrolyzed DOX were released rapidly into tumor cells. Owing to that, the NDDSs exhibited an effective growth inhibition against MDR cells *via* the synergistic effect of ADD and DOX (Wang Q. et al., 2019). On the other hand, Li, Y. et al. reported a sequential release drug delivery system that imparts avoiding the drug efflux and nuclear transport in synchrony *via* a simple nanostructured drug strategy. The liposome-based nanostructured drugs (LNSDs) loaded two modules: DOX loaded into tetrahedral DNA (TD) to form small nanostructured DOX and the nanostructured DOX was encapsulated into the pH-sensitive liposomes. In the *in vivo* experiments, under the weak acidity of lysosomes, liposomes rapidly dissociated and released nanostructured DOX, rapidly increased the intracellular drug concentration, and finally, successfully reduced the drug efflux of MDR and then obtained an ideal therapeutic effect (Li et al., 2019c) (Figure 4A). At the same time, this stimulus can also be used in combination with other stimuli to realize multi-level dissociation of drugs (Li et al., 2019b; Zhang S. et al., 2019; Feng et al., 2020). Zhang, S. et al. engineered a pH/redox dual-responsive NDDS containing DOX and celecoxib (CXB) for synergistically treating drug-resistant breast cancer. This particular kind of NDDS presented significantly accelerated drug release under acidic and redox conditions. In MDR breast cancer cells, this dual-responsive NDDS significantly enhanced the cellular uptake of DOX and the downregulated P-gp expression induced by COX-2 inhibition, and thus obviously increased the cytotoxicity and apoptosis-inducing activity of DOX. Demonstrated by the results of the *in vivo* treatment experiment, nanomedicine remarkably enhanced tumor chemosensitivity and reduced COX-2 and P-gp expressions in tumor tissues (Zhang S. et al., 2019) (Figure 4B).

The gradient change of redox inside and outside tumor cells can be used as another specific stimulus for drug release. Among them, glutathione (GSH) is the most used redox stimulant. This is because the concentration of GSH in the tumor cytoplasm (∼10 mm) is about 7 times higher than that in normal cells (He et al., 2014). Studies have shown that GSH can specifically hydrolyze the disulfide bond and quickly release the drugs covalently connected or dissociate NDDSs. Therefore, the disulfide bond is widely used in the construction of new environmentally responsive NDDSs (Cheng et al., 2011). When this kind of NDDS is exposed to the tumor cytoplasm, it can quickly unload the therapeutic payload through self-decomposition, to maximize its anticancer efficacy and reduce the impact of drug resistance (Maiti et al., 2018; Shen et al., 2018; Wang et al., 2018; Yan et al., 2021). For instance, Yan, J. et al. presented redox-responsive polyethyleneimine (disulfide cross-linked PEI, PSP)/tetrahedral DNA (TDNs)/DOX nanocomplexes (NCs) and PSP/TDNs@DOX NCs to achieve tumor cell/tissue penetration for conquering MDR. The NCs can respond to glutathione and DNase I to disassociate and release DOX rapidly (Yan et al., 2021) (Figure 4C).

#### Construction of NDDSs Actively Targeting the Tumor Lesion

Contributing to the EPR effect, NDDSs are entitled to the ability of passive targeting to tumor tissues, and it is very important to promote the effective and rapid entry of drugs into specific cells (Subramanian et al., 2016). Active targeting provides us with another feasible scheme to enhance the probability of drugs entering specific cells. Active targeting refers to a drug delivery system that covalently connects ligands (with targeting function) to carriers or drug molecules and realizes the connection with target receptors by taking advantage of the special affinity between them. It can enhance the endocytosis of tumor cells and realize the rapid entry of NDDSs into cells. Due to that, it can effectively increase the effective drug concentration in cancer cells and then enhance the curative effect of drugs (Hirsjarvi et al., 2011; Wang et al., 2017; Liu S. et al., 2019; Ma et al., 2019; Soe et al., 2019; Wang Y. et al., 2019; Xiao et al., 2020). For example, based on the high overexpression of Y-1 receptor (Y1R) protein and P-gp in the multidrug-resistant breast cancer cell line, a selective Y1R ligand, [Asn(6), Pro(34)]-NPY (AP) was employed to stabilize the chemotherapeutic drug DOX and P-gp inhibitor tariquidar (Tar) co-loaded nanomicelles at the physiological level. This also improved the targeted delivery of DOX and Tar into MCF-7/ADR cells. Co-delivered Tar further inhibited the efflux of DOX and increased its accumulation in the drug-resistant cancer cells, thereby inducing significant inhibition of cell growth (Wang Y. et al., 2019). Another example is an active targeting nano-sized theragnostic superparamagnetic iron oxide (SPIO) platform for significantly increasing the imaging sensitivity and energy deposition efficiency in the MDR tumor model using a clinical MRgFUS system which was developed by Wang, Z. et al. The surfaces of these PEGylated SPIO nanoparticles (NPs) were conjugated with anti-EGFR (epidermal growth factor receptor) monoclonal antibodies (mAb) for targeted delivery to lung cancer with the EGFR overexpression (Wang et al., 2017) (Figure 4D).

#### Construction of NDDSs Targeting the Site of Drug Action

The therapeutic effect of tumors largely depends on the drug delivery efficiency of NDDSs to its final target, especially for MDR tumors, ensuring the drug concentration near the final target is an important strategy to overcome multidrug resistance. The nucleus has been proved to be the main interaction site of most therapeutics. Therefore, it is highly expected that nuclear targeted NDDSs can provide a more effective strategy than ordinary cell membrane-targeted therapy (Wang et al., 2011; Ling et al., 2012; Li et al., 2018; Li H. et al., 2019; Tu et al., 2020). Tu, Z. et al. conjugated a cyclic R10 peptide [cR(10)] to polyglycerol-covered nanographene oxide for developing a novel nanomedicine to overcome multidrug resistance. The nuclear translocation of this nanomedicine was facilitated by the cR(10) peptide. Subsequently, a laser-triggered release of the loaded DOX results in efficient anticancer activity confirmed by both *in vitro* and *in vivo* experiments (Tu et al., 2020). The biodistribution and photothermal effects of this nanomedicine were studied in HeLa-R tumor-bearing nude mice. These results showed that this nanomedicine could target the tumor lesion most efficiently. At the same time, it has the most photothermal effect under the NIR laser (808 nm), compared to other therapeutic forms. Furthermore, another example is a mesoporous silica nanoparticle (MSN)-based nucleus-targeted system engineered by Li, H. et al., which could directly target the cancer stem cells and further enter the nucleus by the surface modification of anti-CD133 and thermal-triggered exposure of TAT peptides under an alternating magnetic field (AMF). In the *in vivo* treatment of Balb/C mice bearing MCF-7 breast tumor, the nucleus-targeted NDDS efficiently inhibited cancer stem cells by blocking the hypoxia signaling pathway (Li H. et al., 2019).

## NDDSs for Inhibiting DNA Repair

**FIGURE 5:**
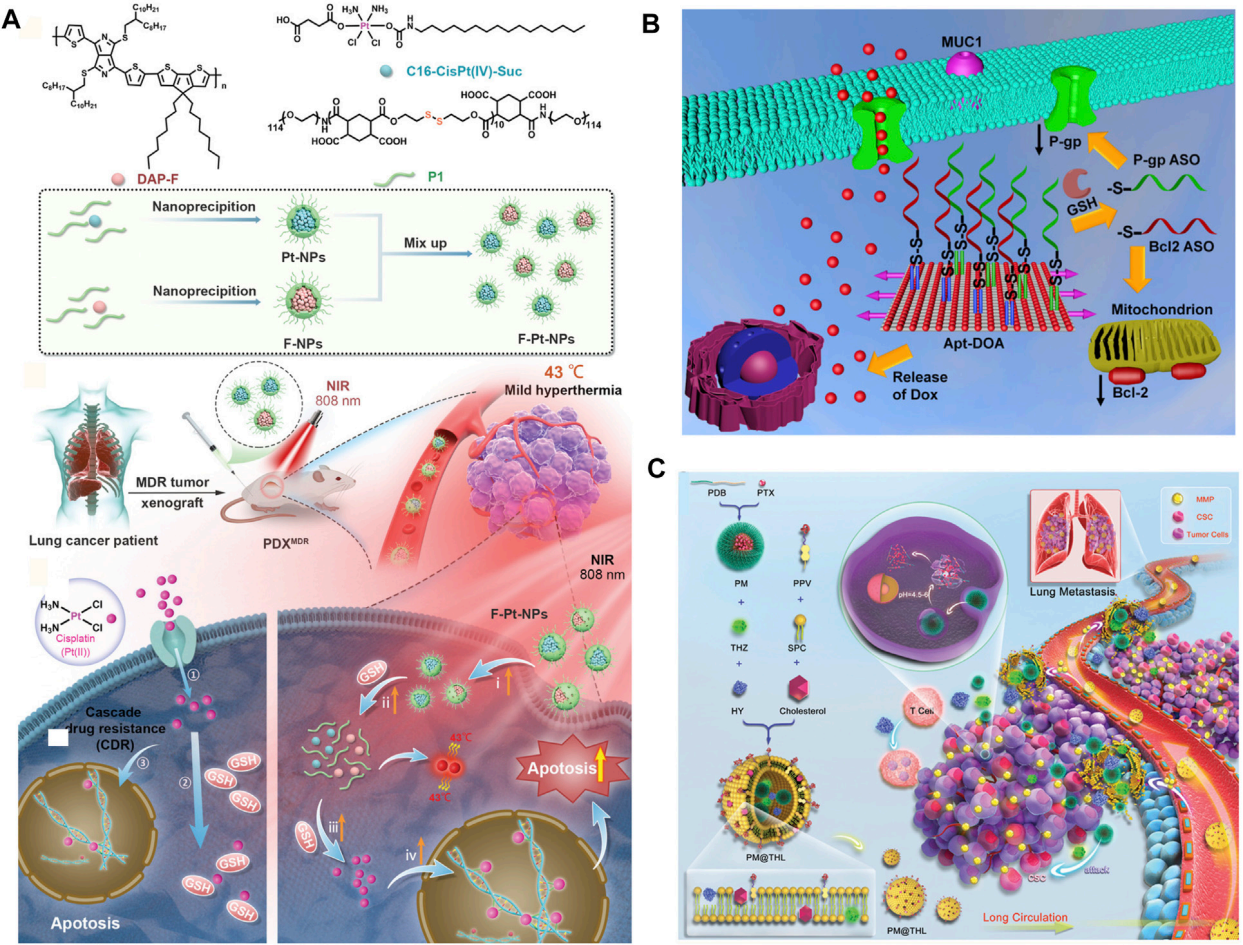
**(A)** Schematic illustration of the preparation of F–Pt-NPs and possible mechanism involved in inhibition of cisplatin resistance under NIR laser irradiation (Wang L. et al., 2021). Copyright 2021 Wiley-VCH GmbH. **(B)** Schematic illustration of the regulation process against MDR tumor of Apt-DOA (Pan et al., 2020). Copyright 2019 American Chemical Society. **(C)** Schematic illustration of components and cocktail therapy of metastatic MDR breast cancer by PM@THL with the dual sensitive double-layered structure (Lang et al., 2019). Copyright 2018 WILEY-VCH Verlag GmbH & Co. KGaA, Weinheim. This figure was generated by Microsoft PowerPoint and OneKeyTools Lite.

In addition to the drug efflux mechanism discussed above, tumor cells can also effectively repair the damaged DNA by excision repair or homologous recombination under the stimulation of chemotherapeutic drugs and eventually weaken or even eliminate the efficacy of chemotherapy (Galluzzi et al., 2012; Housman et al., 2014; Hu et al., 2016). Studies have shown that the process of DNA repair by tumor cells often depends on O6 methylguanine DNA methyltransferase (MGMT), suggesting that MDR caused by DNA repair can be reversed through function or expression inhibition to restore the efficacy of chemotherapeutic drugs, but there were few ideal results at present (Galluzzi et al., 2012; Housman et al., 2014; Hu et al., 2016). Recently, Wang, L. *et al*. proposed a novel systematic combination strategy for overcoming cisplatin resistance using near-infrared (NIR)-light–triggered hyperthermia. The NDDSs, named F-Pt-NP, were composed of two kinds of polymer. Induced by an 808 nm NIR laser, F-Pt-NPs generated mild hyperthermia (43°C) for enhancing the cellular membrane permeability to promote the uptake of drugs and activating cisplatin by accelerating the glutathione consumption. Moreover, it could increase the Pt-DNA adduct formation and possibly the formation of a portion of irreparable Pt-DNA interstrand crosslinks, which significantly inhibited the repair of DNA. *In vivo*, on a patient-derived xenograft model of multidrug-resistant lung cancer (A549DDP), the efficacy of the F-Pt-NPs treatment group showed a tumor inhibition rate of 94% (Wang L. et al., 2021) (Figure 5A). Similarly, Xin, J. et al. reported a carrier-free aquo-cisplatin arsenite multidrug nanomedicine loaded with cisplatin and arsenic trioxide prodrugs simultaneously. This nanomedicine achieves a high loading capacity and pH-dependent controlled release of the drugs. Cisplatin and arsenic trioxide in this nanocomposite can induce massive DNA damage and inhibit the activity of PARP-1, which is closely related to the DNA damage repair in cisplatin-resistant tumor cells. Gene expression profiles demonstrated the expressions of proto-oncogenes, and DNA damage repair–related genes *MYC*, *MET*, and *MSH2* were reduced, respectively. On the other hand, the expressions of tumor suppressor genes *PTEN*, *VHL*, and *FAS* were increased. This nanocomposite showed strong cytotoxicity against cisplatin-resistant ovarian tumor cells and could overcome cisplatin resistance effectively (Xin et al., 2019).

## NDDSs for Reactivating the Apoptosis Pathway of Tumor Cells

The reactivation of the anti-apoptotic process of tumor cells plays another important role in resisting the attack in chemotherapeutics, which involves promoting survival, anti-apoptotic regulator Bcl-2, and its downstream nuclear factors-κB (NF-κB), and so on. More than 50% of cancers have defects in the mechanism of apoptosis. The most characteristic of these abnormalities is the increased expression of Bcl-2 family proteins (Housman et al., 2014; Hu et al., 2016). Targeting these anti-apoptotic factors to improve the killing efficiency of chemotherapeutics against tumor cells is a promising tumor therapy strategy (Pan et al., 2020; Wang H. et al., 2020; Xie et al., 2020; Sivak et al., 2021). For instance, Sivak, L. et al. described a polymer biomaterial composed of the antiretroviral drug ritonavir derivative (5-methyl-4-oxohexanoic acid ritonavir ester; RD), covalently bound to the HPMA copolymer carrier *via* a pH-sensitive hydrazone bond (P-RD). Importantly, RD inhibited STAT3 phosphorylation in CT26 cells (murine colon adenocarcinoma) *in vitro* and the expression of the NF-κB p65 subunit, Bcl-2, and Mcl-1 *in vitro*. P-RD nanomedicine showed significant antitumor activity in CT26 and B16F10 tumor-bearing mice (Sivak et al., 2021). Additionally, Pan. Q. et al. constructed a multifunctional DNA origami-based nanocarrier for co-delivery of a chemotherapeutic drug (doxorubicin, DOX) and two different antisense oligonucleotides [ASOs; B-cell lymphoma 2 (Bcl2) and P-gp] into drug-resistant cancer cells for enhanced therapy. Experiments revealed that the origami could protect ASOs against nuclease degradation in 10% FBS. Moreover, with the synergetic effect of co-delivery of multi-ASOs and DOX, the anticancer assay showed that Apt-DOA (Apt-DOX-origami-ASO) could circumvent multidrug resistance and significantly enhance cancer therapy in Hela/ADR and MCF-7/ADR cells (Pan et al., 2020) (Figure 5B).

## Accurately Targeting Cancer Stem Cells and Improving Reversal Efficiency

Recent studies have shown that a small number of tumor cells have the characteristics of stem cells and usually have drug resistance ability, due to the heterogeneity of tumor tissues (Housman et al., 2014). In addition, another small number of adult tumor cells also have the same ability (Housman et al., 2014). In conventional tumor treatment, these drug-resistant tumor cells can survive and continue to proliferate as seeds over time, leading to recurrence (Housman et al., 2014). Some of these drug-resistant cancer cells may enter the blood circulation and may form metastases in distant organs (Mansoori et al., 2017; Bukowski et al., 2020). Therefore, through the rational design and construction of new NDDS, accurately targeting cancer stem cells or adult cancer cells that have developed drug resistance can effectively improve the efficiency of reversing multidrug resistance and inhibiting tumor growth. As discussed before, the cancer stem cell-specific–targeted mesoporous silica (mSiO_2_)-dendritic polyglycerol (dPG) nanocarriers co-delivered the chemotherapy drug DOX and the P-gp inhibitor tariquidar (Tar) for reversing MDR and enhancing chemotherapy to breast cancer stem cells (Pan et al., 2021). In addition to the active targeting strategy, relying on the intelligent change of nanomedicine size for deeply penetrating the tumor tissue and improving the clearance of cancer stem cells is also an important strategy for reversing MDR efficiently. Based on that, a particular kind of morphology-tunable nanomedicine was developed. Chemotherapy and immune checkpoint blocking therapy for large tumor cells and cancer stem cells were integrated into a drug delivery system. The particle size shrank when the nanoparticle transferred from circulation to tumor tissues, favoring both pharmacokinetics and cellular uptake, meanwhile achieving the sequential drug release where needed. This nanomedicine reduced the proportion of cancer stem cells and enhanced the therapeutic efficacy against the tumor and thus prolonged the survival time of mice (Lang et al., 2019) (Figure 5C).

## Discussion

With the continuous development of nanotechnology, the application of NDDSs in cancer treatment has received extensive attention all over the world. In fact, according to the NIH, there are 110 clinical trials involving the application of nanotechnology for cancer treatment nowadays (Gonzalez-Valdivieso et al., 2021). Among them, Doxil^®^ is a representative example, which is the first FDA-approved NDDS for clinic application since 1995, consisting of PEG-liposome and doxorubicin. Compared to free doxorubicin, Doxil^®^ has some important advantages, such as a 100-times longer half-life inside the body circulation and a reduction in cardiotoxicity. Due to that, Doxil^®^ has been applied in several clinical therapies of malignant tumors, such as myeloma and ovarian cancer (Barenholz, 2012). Similarly, another FDA-approved NDDS is Abraxane^®^, which is composed of albumin particles and paclitaxel. The results of clinical studies showed that, compared with Taxol^®^, Abraxane^®^ can significantly reduce the toxic side effects caused by Cremophor^®^ in Taxol^®^ and increase the maximally tolerated dose of patients by 80% (Green et al., 2006; Desai, 2016).

In addition to these, other novel NDDSs, which are constructed based on different strategies, have entered the clinical research from preclinical research in the laboratory, including passive-targeting tumor lesions *via* the EPR effect or active-targeting tumor sites relying on the antibody technology, such as PK2, an HPMA polymer doxorubicin conjugate, was the first targeting nanotherapeutic agent reaching the clinic (Julyan et al., 1999; Hopewell et al., 2001; Kopeček and Kopečková, 2010; Gonzalez-Valdivieso et al., 2021). Notably, in addition to the primary tumor-targeting NDDSs, some NDDSs aiming at reducing tumor metastasis have also been gradually translated into clinical studies, such as DaunoXome^®^ which was employed for the treatment against metastatic ovarian cancer and metastatic breast cancer (Eckardt et al., 1994; Forssen, 1997). Unfortunately, for patients with MDR, although in preclinical laboratory studies, researchers have developed many potential NDDSs described above, which can obtain exciting *in vivo/vitro* therapeutic effects in animal models, especially mouse models; there is still no NDDS for reserving MDR that has been transferred into the clinic due to the great differences between human and animal models, as well as the significant challenges brought by the complex mechanism of MDR to the construction strategy of NDDSs.

Thus, novel strategies for overcoming these following obstacles that limit the clinical translation process of NDDSs are needed. First, although nanoscale carriers can enhance drug penetration and accumulation within tumor lesions, the actual number of drugs that can eventually enter tumor tissues is still limited due to the generally low drug content in NDDSs, and the vehicles themselves have clinical risks (Stiegler et al., 2010; Yang et al., 2019; Chen S. et al., 2020). At present, some nanoscale carriers are related to oxidative stress, adverse inflammatory reaction, and genotoxicity (Liu et al., 2013; Zuo et al., 2013; Yuan et al., 2020). Based on this, researchers began to focus on the development of carrier-free NDDSs. For example, siRNA itself was used as the carrier for drug delivery by using gene origami technology and a kind of carrier-free NDDS composed of the drug-chemogene conjugate. This drug-chemogene was formed by two paclitaxel (PTX) molecules with a floxuridine (FdU)-integrated antisense oligonucleotide (terminated chemogene), which take fluorescent dithiomaleimide (DTM) as a linker. This carrier-free drug delivery system obtained the ability to knock down the expression of P-gp and the synergistic inhibitor effect (Zhu et al., 2020). Similarly, we recently combined the COX-2 inhibitor indomethacin (IND) with the chemotherapeutic drug paclitaxel through a simple self-assembly to obtain a series of nanomedicine with different morphologies and achieved ideal results in preliminary antitumor therapy (Zhang C. et al., 2019). Because COX-2 inhibitors can effectively inhibit the production of ATP, this carrier-free NDDS has the potential to reverse tumor multidrug resistance and was of great value for further research. The above research can not only effectively reduce the use of inactive carrier materials and effectively improve the drug content but also greatly reduce the preparation difficulty of NDDSs and then has a broader prospect of clinical transformation. However, how to rationally integrate the existing concept of developing NDDSs for multidrug-resistant tumors with carrier-free NDDSs is still an urgent problem to be solved by researchers, which needs to make great efforts.

In addition, because the mechanism of MDR is very complex and changeable, simply targeting one mechanism is far from meeting the clinical needs. Therefore, exploring a reasonable drug combination to achieve multi-channel reversal of multidrug resistance and tumor treatment is a further research direction in this field. At present, researchers have made some corresponding exploration. For instance, Liu, X. et al. developed a novel kind of gold nanoparticle (AuNP) modified by multifunctional molecular beacons (MBs), which was utilized as a vehicle for loading three different drug resistance–related mRNAs (MDR1 mRNA, MRP1 mRNA, and BCRP mRNA). After uptake by cells, MBS AuNP would inhibit the translation of drug resistance–related mRNAs and reduce the flux protein expression. This NDDS was provided to have the ability of synergistic inhibiting MDR and *in situ* imaging of drug resistance–related mRNAs in living cells (Liu X. et al., 2019). Delivering drugs by passing the drug efflux mechanisms of MDR is also a solution to those problems discussed above. For example, telomerase, as a biomarker of tumor cells, is expressed in more than 90% of tumor cells. The inhibition of telomerase can effectively increase the chemosensitivity of tumor cells. Therefore, the construction of new NDDS-targeting telomerase has also achieved an obvious therapeutic effect on multidrug-resistant tumors (Wu Y. et al., 2020).

In conclusion, even though key steps are still needed to improve their possibility of clinical translation, NDDSs are becoming more and more promising to eventually solve MDR of tumors in clinical application. Based on this fact, we have reason to believe that in the foreseeable future, there will be an NDDS that can effectively solve the MDR of tumors and be translated into clinical application.
